# Measuring Burden of Disease Attributable to Air Pollution Due to Preterm Birth Complications and Infant Death in Paris Using Disability-Adjusted Life Years (DALYs)

**DOI:** 10.3390/ijerph17217841

**Published:** 2020-10-26

**Authors:** Séverine Deguen, Guadalupe Perez Marchetta, Wahida Kihal-Talantikite

**Affiliations:** 1 EHESP School of Public Health, Rennes Campus, CEDEX 35043 Rennes, France; guadalupe.perezmarchetta@eleve.ehesp.fr; 2 Department of Social Epidemiology, INSERM, Institut Pierre Louis d’Epidémiologie et de Santé Publique (UMRS 1136), Sorbonne Universités, UPMC University Paris 06, 75012 Paris, France; 3 LIVE UMR 7362 CNRS (Laboratoire Image Ville Environnement), University of Strasbourg, 6700 Strasbourg, France; wahida.kihal@live-cnrs.unistra.fr

**Keywords:** environmental burden of disease, years life lost, disability-adjusted life years, air pollution, adverse birth outcome

## Abstract

Several studies have found maternal exposure to particulate matter pollution was associated with adverse birth outcomes, including infant mortality and preterm birth. In this context, our study aims to quantify the air pollution burden of disease due to preterm birth complications and infant death in Paris, with particular attention to people living in the most deprived census blocks. Data on infant death and preterm birth was available from the birth and death certificates. The postal address of mother’s newborn was converted in census block number. A socioeconomic deprivation index was built at the census block level. Average annual ambient concentrations of PM_10_ were modelled at census block level using the ESMERALDA atmospheric modelling system. The number of infant deaths attributed to PM_10_ exposure is expressed in years of life lost. We used a three-step compartmental model to appraise neurodevelopmental impairment among survivors of preterm birth. We estimated that 12.8 infant deaths per 100,000 live births may be attributable to PM_10_ exposure, and about one third of these infants lived in deprived census blocks. In addition, we found that approximately 4.8% of preterm births could be attributable to PM_10_ exposure, and approximately 1.9% of these infants died (corresponding to about 5.75 deaths per 100,000 live birth). Quantification of environmental hazard-related health impacts for children at local level is essential to prioritizing interventions. Our study suggests that additional effort is needed to reduce the risk of complications and deaths related to air pollution exposure, especially among preterm births. Because of widespread exposure to air pollution, significant health benefits could be achieved through regulatory interventions aimed at reducing exposure of the population as a whole, and particularly of the most vulnerable, such as children and pregnant women.

## 1. Introduction

The fact that preterm birth is associated with increased neonatal morbidity and mortality is now well established. The main complications of preterm birth include both several morbidity in adulthood and neurodevelopmental impairments that may, for instance, increase the risk of cerebral palsy which affects long-term physical health [1]. Moreover, preterm birth is known to be an important risk factor for infant death [2]. The World Health Organization (WHO) cited preterm birth complications as a leading cause of death among children under five in 2017 [3]. In 2010, Blencowe et al. estimated that about 15 million babies were born preterm, representing 11.1% of all live births worldwide [4]; this percentage falls to about 5% in European countries [5]. When we take into account diminished quality of life, consequences for the family’s life and costs related to health care, preterm birth clearly represents a major burden. In the United States, Behrman and Butler found the annual economic burden associated with preterm birth to be estimated as at least $26.2 billion USD ($51,600 USD per infant)—approximately 65% of which was attributed to medical care [1].

In addition, several studies found particulate matter pollution exposure to be associated with adverse birth outcomes, including infant mortality and preterm birth. Recent meta-analysis confirms that the risk of post-neonatal mortality all-causes for short-term exposure to PM_10_ increased significantly (pooled—OR = 1.013, 95% CI = 1.002–1.025) [6]. In addition, Klepac et al., revealed that exposure to particulate matter over the entire pregnancy was significantly associated with higher risk for preterm birth (the pooled effect estimates were 1.09 (1.03–1.16) per 10 μg/m^3^ increase in PM_10_ [7].

The World Health Organization has identified air pollution as a leading cause of the environmental burden of disease in the European Region [8], estimating that in France, 14% of the total DALYs (disability adjusted life years, DALYs) can be attributed to environmental factors [9], with about 4800 premature deaths per year attributable to PM_10_ exposure [10].

Assessment of environmental burden of disease estimates therefore offers an opportunity to identify policy priorities, particularly among newborns—a population recognized as more vulnerable to its living environment. An estimation of how many infant diseases and deaths are caused by air pollution constitutes a starting point with which to guide policymakers in drawing up infant and child health protection policies and measures. For policymakers, disease burden estimates can provide crucial information on the health benefits that could be achieved through the implementation of targeted actions against specific risk factors. With rising interest in the development of interventional research into public health issues, a crucial goal seems to be a design study quantifying what might be the “best” environmental intervention, in terms of cost and health benefits.

To date, few studies [11] have estimated the environmental burden of disease related to preterm birth due to the paucity of gestational age-specific data. Indeed, preterm birth complications are clearly inversely related to gestational age and must be integrated to calculation of the disability adjusted life years (DALYs). To our knowledge, no French study has been conducted using input data estimated at the fine scale in assessment of the environmental burden of disease due to preterm birth and infant death in order to capture census block variability. These interventions aim to improve living environments by decreasing exposure to environmental nuisances. In this context, our study aims to quantify the air pollution burden of disease due to preterm birth complications and infant death in Paris, using the Disability-Adjusted Life Years indicator.

## 2. Materials and Methods

### 2.1. Study Area

The study area is Paris, the capital of France. The number of inhabitants is about 2,250,000 distributed into 992 census blocks. It corresponds to 2199 inhabitants per census block on average and to a population density about 21,428 inhabitants per km^2^. The city of Paris constitutes an appropriate place in which to investigate this issue, since average air pollution levels exceed the WHO guidelines. In addition, the population size (with around 30,000 newborns per year) is high enough to calculate both Years of Life Lost (YLL) and disability adjusted life years (DALYs) from a substantial preterm birth and infant death sample.

### 2.2. Health Databases and Outcomes Definition

Two different health databases were used in this study. First, infant death data was collected from death certificates recorded at Paris City Hall over the period between 2004 and 2009 [12]. All cases were geocoded to the census block of the postal address at birth. In addition to the postal address of the parent, the only variable available from the death certificate was the date of death. Second, we considered all singleton births in Paris between 2009 and 2011 from the birth certificate information registered by the Maternal and Child Care department of Paris (Protection Maternelle et Infantile, known as PMI) [13]. This is a certificate completed by parents and health professionals prior to discharge from the maternity ward, then sent to the PMI for the department of residence. This certificate provides a set of information about the newborn and his/her parents; for this study specifically, we considered gestational age (in weeks) in order to define preterm births (<37 weeks). All maternal postal addresses were geocoded at census block level. Because of their slim chance of survival, we excluded extremely preterm babies (gestational age <20 weeks) from our analysis. In addition, for these babies, the risk of death related to air pollution is very weak compared to other individual risk factors.

### 2.3. Ethical Approval

Ethical approval was obtained from the French Data Protection Authority (CNIL). For reasons of confidentiality, in order to comply with the ethical authorization provided for this study (authorization numbers 914118 and 911149), it was not possible to store the individual localization of families.

### 2.4. Census Block Neighborhood Socioeconomic Level

Census block socioeconomic level was assessed using a composite deprivation index. This composite index captures various socioeconomic dimensions: Family structure, household type, immigration status, employment, income, education and housing. The data for Paris is available from the National Institute of Statistics and Economic (INSEE—2012) [14]. Principal Component Analysis was used to select those socioeconomic variables most correlated with the first principal component, and then to calculate the socioeconomic deprivation value of each census block (further methodological details are given in Lalloué et al. [15]. This deprivation index was categorized into 5 classes of census block, according to the quintile of its distribution.

### 2.5. Air Pollution Measurement

A many epidemiological studies, toxicological evidence and WHO review supported that ambient Particulate Matter (PM) is considered responsible for the health effects [16,17]. Therefore, we chose the most reasonable indicator PM_10_ for which newborn health effect are well documented and dose–response function available.

AirParif is the local association, which monitor the air quality in Paris. Based on ESMERALDA Atmospheric Modeling system [18] they modeled average annual ambient concentrations of PM_10_ at census block level from January 2007 to December 2012. The model integrates integrate meteorological data: Air temperature, wind speed and direction, relative humidity, barometric pressure (supplied by Météo France, the French meteorologic service), emission sources according to their contribution to ambient air pollution and background pollution measurements as input parameters. Selected emission sources were linear sources (main roads), surface sources (diffuse road sources and residential and tertiary emissions) and important point sources [18].

### 2.6. Statistical Methods

Our statistical analysis was structured as follows:

First, we described our study population in terms of infant death rate and preterm birth rate by specific gestational age group, and both health events by neighborhood socioeconomic classes.

Second, we used the health impact assessment approach to calculate the number of infant deaths attributable to PM_10_ between 2004 and 2009 and the proportion of deaths for preterm births between 2009 and 2011. Next, the number of attributable deaths was converted to YLL.

Third, we used the three-step compartmental model to estimate total Years lost Due to Disability (YLD) among preterm births, and the proportion of YLD attributed to PM_10_ exposure.

Lastly, we presented the results by quintile of the neighborhood socioeconomic index distribution. This step aims to reveal the potential existence of unequal YLL and YLD distribution by neighborhood socioeconomic class.

#### 2.6.1. Estimation of Number of Attributable Health Events

We applied a Health Impact Assessments (HIAs) to estimate the number of health events (infant death and preterm births, respectively) associated with a reduction in PM_10_ concentrations in Paris, using the counterfactual method [19].

This approach requires several data sources:

(i) Population size (in this study, the number of live births per year among residents of Paris) and level of exposure (population exposure),
(ii) baseline of infant death rate and preterm birth rate,
(iii) dose–response function derived from meta-analysis, which estimated the meta-relative risk, associated with the increase of PM_10_ exposure [20]. In our study, number of health events that could be attributed to PM_10_ exposure were assessed for hypothetical air pollution reductions in line with WHO recommendations (for PM_10_, it was 20 μg/m^3^ called the counterfactual value), using the following Equation (1).

(1) $$\Delta Y=Y0\times (1-e^{-\beta \times \;\Delta x})$$

where:

- Y_0_ corresponds to the total number of observed infant deaths or preterm births,
- Δx measures the difference between the observed average of the PM_10_ and the guideline value of WHO, and,
- β is the natural logarithm of meta relative risk expressed for an increase of 10 µg/m^3^ of PM_10_

We used two different meta-risks arising out of the literature. Increased risk of preterm birth related to an increase of 20 µg/m^3^ of PM_10_ concentrations is equal to 1.05 (95%CI = 1.02–1.07) [21]. Risk of death among the infant population is 1.04 (95% CI = 1.02–1.07) for an increase of 10 µg/m^3^ in PM_10_ concentrations [22].

All input data used in our study were describe in the Table 2.

#### 2.6.2. Calculation of Years of Life Lost (YLL) Attributed to Infant Mortality

The number of infant death associated with decreased PM_10_ can be converted to a number of Years of Life Lost (YLL) by using this equation:

YLL = N × L(2)

where:

- N is the number of deaths
- L is the standard life expectancy in years

In Paris, average life expectancy for the period 2004–2009 was 82.8 years [23].

#### 2.6.3. Calculation of Disability-Adjusted Life Years (DALYs) Attributed to Preterm Birth

To measure the burden of disease as a result of neurodevelopmental impairment due to preterm birth, we estimated the prevalence-based DALY, which incorporates YLL (Years of Life Lost) and YLD (Years lost Due to Disability), as defined by the following formula:

DALY = YLL + YLD(3)

where:

- YLD = P × DW
- P is the number of prevalent cases, and
- DW is the disability weight

**Figure 1:**
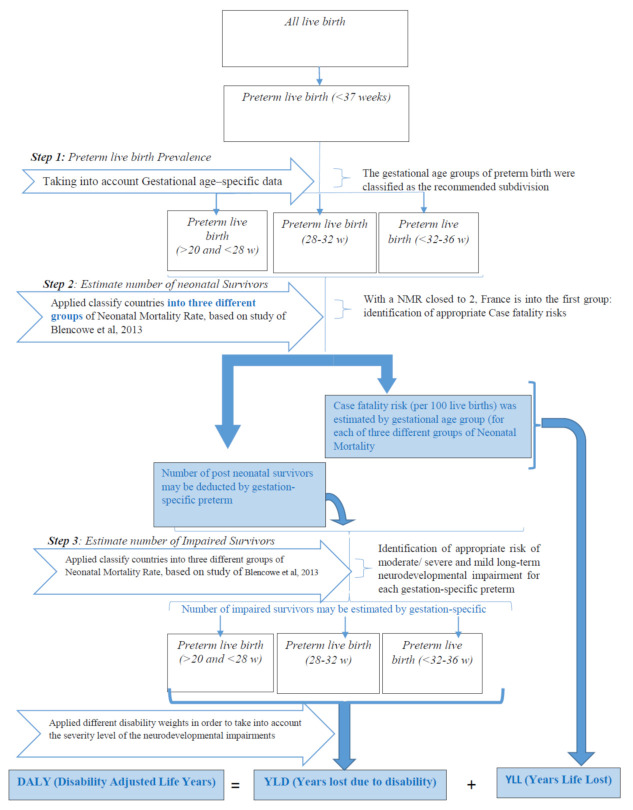
Descriptive of DALYs (disability adjusted life years) steps.

Figure 1 describes the whole process followed in order to estimate Disability-Adjusted Life Years (DALY) attributed to preterm birth.

We used the three-step compartmental model proposed by Blencowe et al., with estimated input parameters being used to assess neurodevelopmental impairment among post-neonatal survivors of preterm birth [24]. In the first step, we estimated the prevalence of preterm birth among live births. The number of neonatal survivors, and the proportion of these people having potential impairments, were estimated in steps 2 and 3 respectively. We describe each step below.

Firstly, we estimated live birth prevalence among preterm births. Since decreasing gestational age is associated with increasing mortality, disability, and a greater requirement for intensive neonatal care, gestational age of preterm birth was classified as the recommended subdivision: Extremely preterm birth (<28 weeks and >20 weeks), moderate preterm birth (28–32 weeks), and late preterm birth (32–36 weeks). The gestational distribution of preterm birth attributed to PM_10_ was assumed to follow the same distribution for all births.

Secondly, we estimated number of Post-Neonatal Survivors. This step allowed us to estimate both post-neonatal survivors and number of preterm deaths, by gestational age group. As Blencowe et al. have already explained, a correlation exists between health system factors in place in each country and the neonatal mortality rate [24]. They therefore suggested classifying countries into three different Neonatal Mortality Rate (NMR) groups, as follows: Low rate (<5), moderate rate (5 to 15) and high mortality rate (≥15). With an NMR close to 2, France is in the first group. Based on a population of 388,253 preterm births in 21 different countries, the case fatality risk (per 100 live births) was estimated by gestational age group. Therefore (where we know the corresponding NMR), the number of post-neonatal survivors can be deducted by gestation-specific preterm group. Case fatality risks are summarized in Table 1. The number of preterm deaths by gestational age group estimated at this step has allowed us to calculate the YLL attributed to preterm birth.

Finally, we estimated number of Impaired Survivors. This step aims to estimate the number of impaired survivors, based on the risk of moderate/severe and mild long-term neurodevelopmental impairment after preterm birth. Table 1 gives the risk estimates by gestation-specific preterm.

#### 2.6.4. Disability Weights

As shown in Equation (3), estimation of DALYs relies on assessing prevalence using disability weights. In our study, in order to take into account the severity level of neurodevelopmental impairments, we distinguished two distinct disability weights. The disability weight used for moderate-to-severe neurodevelopmental impairment was 0.38 (uncertainty range: 0.29–0.49). Assuming that about 50% of those with mild impairment had isolated mild problems, and 50% had mild motor and mild cognitive impairment, the disability weight used for these people was 10 fold lower, at 0.03 (uncertainty range: 0.02–0.05) [24]. Table 2 gives a summary of all the input data.

## 3. Results

### 3.1. Baseline Health Data and PM_10_ Data

The mean concentration of PM_10_ between 2007 and 2012 was 30.1 µg/m^3^ (Standard Deviation = 1.72 μg/m^3^). The rate of infant death was 3.36 per 1000 live births over the period 2004–2009 (Total number of births approximately equal to 187,450 over the period 2007–2012), while the rate of preterm birth reached 6.1% between 2009 and 2011 (Total number of births = 86,877). The number of preterm births among all births is equal to 0.22%, 0.62% and 5.34% at less than 28 weeks (and >20 weeks), at 28–31 weeks and at 32–36 weeks, respectively. We also estimate that 3.6% of all preterm births were at less than 28 weeks (and >20 weeks); 10.0% at 28–31 weeks and 86.4% at 32–36 weeks.

The trend test reviled that the death rate per 1000 live births increased significantly with the level of neighborhood socioeconomic deprivation (*p* < 0.016). The death rate is 1.9 fold higher among newborns living in the most deprived census blocks of Paris than among those living in the least deprived census blocks (Table 3).

The percentage of preterm births differs significantly between neighborhood socioeconomic classes across all levels of preterm: Extremely preterm (*p* < 0.029), moderate preterm (*p* < 0.001), and late preterm (*p* < 0.002). More precisely, we found that the percentage of extreme preterm is 1.9 fold higher among newborns living in the most deprived census blocks than among those living in the least deprived census blocks (Table 4). These ratios are lower among the moderate and late preterm births, equal to 1.41 and 1.11, respectively.

### 3.2. DALYs for Preterm Birth Complications

Applying the case fatality risks (CFR) function by gestational-specific preterm birth category (20–28, 28–32, and 32–36 weeks), we found that 118 died among 5363 preterm infants born in Paris between 2009 and 2011. This corresponding to about 1.36 per 1000 live births, which contributed to 9770 YLL. Knowing that the infant death rate was estimated at 3.36 per 1000 live births, about one third of these infants would be born preterm. We estimated that about 46% of deaths were of infants born in the extreme preterm period. YLD value was 71.3 including both severe/moderate and mild long-term neurodevelopmental impairments. The DALY value from preterm birth complications was 9841.3.

### 3.3. Burden of Disease Attributed to PM_10_ Exposure

Of the 629 infant deaths occurring during the period 2004–2009, we quantified that 24 (95% CI = 12–43) deaths were attributable to PM_10_ exposure, equivalent to 1987.2 years of life lost: It corresponds to about 12.8 deaths per 100,000 live births, and 1049.9 YLL per 100,000 live births.

Of the 5363 preterm births counted among all births in Paris (2009–2011), we estimated that 258 (95% CI = 106–354) maybe attributable to PM exposure. Of the preterm birth infants who died, 5 (95% CI = 2–8) were attributed to PM_10_ exposure (equivalent to 414 YLL). This also corresponds to 5.75 deaths per 100, 000 live births. The number of preterm births attributed to PM_10_ exposure and living with long-term neurodevelopmental impairment was equal to 71.1 (95% CI = 66.0–77.6).

### 3.4. Unequal Social Distribution

In terms of preterm birth complications, we found that about 13% of infant deaths occurred in families living in the least deprived census blocks, whereas this percentage increased to 20% for those living in the most deprived census blocks. Concerning deaths attributed to PM_10_ exposure, we observed that about 1/3 of the deaths occurred in families living in the most deprived census blocks; however, to due to the very low number, it is impossible to formulate a general conclusion from this observation.

## 4. Discussion

The WHO states that DALY is traditionally used in the quantification of the burden of disease, to estimate the magnitude of health loss due to diseases and risk factors for specific populations at a given age. To our knowledge, our study is one of the first to quantify the health consequences of PM_10_ exposure estimated at a fine spatial scale among a newborn population; thus, it is difficult to make literature comparison.

We estimated that 12.8 infant deaths per 100,000 live births may be attributable to PM_10_ exposure. Our finding is coherent with a US study published in 2004 [26], whose risk assessment study suggested that outdoor air pollution above a threshold fixed at 12 µg/m^3^ PM_10_ would contribute in a substantial way to post-neonatal infant mortality: About 14.7 infant deaths (95% CI = 7.3–25.6) per 100,000 live births.

In a study recently conducted in Kuwait, 17 excess death cases may be attributable to PM_10_ exposure, which is equivalent to 53.77 (95% CI = 28.87–84.95) per 100,000 live births [27].

However, it is difficult to make comparisons with our findings, as their current average of PM_10_ was equal to 167.5 mg/m^3^ and the threshold used was 70 mg/m^3^.

The difference between the result of our study and the previous studies may be related to several reasons.

First, there was a difference in the definition of outcome between the studies. The US and Kuwait studies analyzed post-neonatal mortality (between 28 days and 1 year) [26,27], More specifically, US study estimated more specific death causes such as sudden infant death syndrome and respiratory disease mortality among normal birth weight [26]. Whereas in our study, we estimated the infant death due to PM_10_ exposure among all live births (<1 year).

The second difference in the method of assessment of environmental burden of disease is related to the choice of Dose response function. In US study, the environmental assessment were of the two studies based on the only currently available cohort US study [26]. In the Kuwait study, the authors used the relative risk define by the World Health Organization (WHO) regional office for Europe (Bonn office, Germany) in the AirQ+ model [20]. In our study, we used a dose–response function derived from meta-analysis.

The third difference between studies is related to environmental data used in the assessment approach. The US study [26] and Kuwait study [27] used annual mean outdoor PM_10_ levels from the monitoring station data, whereas in our study, we used modeled average annual ambient concentrations of PM_10_.

Another reason for this study to be different is the spatial level of input data included in the assessment of environmental burden of disease. While in our study we estimated the infant death attributed to PM_10_ exposure measured at the census block level, in the US study [26] and Kuwait study [27] attributable mortality to PM_10_ estimated at counties level.

Our study also revealed that the YLL value from preterm birth complications (independent of air pollution exposure) was about 11,245.8 YLL per 100,000 live births. A recent study conducted in Korea found that about 18,961.2 YLL were related to preterm birth. The Korean estimate is in the same order of magnitude as ours; however, it is not directly comparable because of the high preterm birth rate in Korea, which ranged from 13.5% and 15.7% between 2008 and 2013, compared to Paris where the rate is about two times lower [11].

In addition, due to PM_10_ exposure, we estimated that about 4.8% of preterm birth could be attributable to PM_10_ exposure, while about 1.9% of these infants died (corresponding to about 5.75 deaths per 100,000 live births). In a US study, Trasande et al. found that 3.32% of preterm births reported in 2010 could be due to PM_2.5_ (PM_2.5_ > 8.8μg/m3). They also explained that preterm births also have emotional and psychological consequences for parents and families, which need to be considered in the total benefits assessment [28]. A global, regional and national assessment of preterm birth associated with PM exposure was conducted in 2017; it estimated that in western Europe, about 19% and 38.9% of total preterm births may be attributable to PM_2.5_ exposure, with a low concentration cut-off set at 10 μg/m^3^ and at 4.3 μg/m^3^, respectively [29]. Another European study suggested that a reduction in PM_2·5_ concentration to 10 μg/m^3^ during pregnancy would reduce low birthweight at term by 22% (95% CI = 8–33) [30].

We have shown that both infant deaths and preterm births increased significantly with the level of neighborhood socioeconomic deprivation. We also suggest a possible increase in health burden related to PM_10_ exposure. However, the study period was not long enough to count an adequate number of health outcomes per neighborhood socioeconomic class. The statistical power is too low to show a significant increase; for this reason, we cannot rule out the possibility that our observation could be put down to chance.

The main strength of our study is linked to the quality of the input data. The advantage of the health data used in our study is the rate of completeness of the birth certificate, which reached 93% on average, coupled with the large population size, resulting in a small variability of our estimates [31]. The air pollutant modelling procedure used provides unbiased estimates of exposure to PM10. This type of model was validated by Jerrett et al., who demonstrated its effectiveness and reliability [32]. However, the interpretation of our findings must also consider certain weaknesses. The limitations of the present study are related to both assessment of exposure level to PM_10_ attributed to each newborn and health data.

One main limitation of our work is the uncertainties related to the assumptions that air pollutant exposure during pregnancy would increase significantly the adverse birth outcome including preterm birth and infant mortality. This hypothesis rise two main weakness:

(i) The generalization of this evidence. Many recent studies, including meta-analysis [7] have concluded that air pollution may have adverse pregnancy outcomes such as preterm birth, small head circumference at birth, as well as neonatal and post-neonatal mortality. For instance, Pedersen et al. found significant association between PM_10_ and NO_2_ exposure during pregnancy and the risk of low birthweight at term PM_10_ (OR for 10 μg/m^3^ increase 1·16, 95% CI = 1.00–1·35), NO_2_ (OR for 10 μg/m^3^ increase 1·09, 1·00–1·19) [30]. In European country, Schifano et al., found consistently in Rome and Barcelona, an increased risk of preterm birth (in a more pronounced way among the early preterm birth) for a unit increase in PM_10_ and NO_2_ [33]. In addition, a large population-based prospective cohort study conducted in Rotterdam found that maternal exposure to PM_10_ and NO_2_ is inversely associated with fetal growth and with weight at birth. The authors revealed that only PM_10_ exposure levels were positively associated with preterm birth and Small Gestational Age (SGA) [34]). Even scientific evidences tend to reveal that the risk of adverse birth outcomes increase with the increase in air pollution level during pregnancy, several studies did not confirm this finding. For instance, European meta-analysis of 13 cohort studies did not found association between risk of preterm birth and atmospheric pollutants [35]). However, as the authors discussed, they did not take into account information on maternal, fetal and placental conditions to identify preterm birth cases with different proximal etiology.
(ii) Evidences for causality. It is also important to recall the existence of uncertainty in any epidemiological studies (detailed above) suggesting (or not) significant association between air pollution exposure and birth outcomes. This uncertainty may affect the causal relationships or the shape of the exposure–response relation. Thus, due to these uncertainties may bias the input data of the Environmental Burden of Disease (EBD) and turn in uncertain assessment of DALYs, which have to be interpreted with caution.

Finally, an additional limitation, also source of uncertainty, is related to the geographic extrapolation of the dose–response function derived from international meta-analysis.

We have not attempted to use only one cohort study for a meta-analytic estimate because we considered that differences in study regions do not allow transferring results.

Then, we hypothesis increased risk of preterm birth related to an increase of 20 µg/m^3^ of PM_10_ concentrations is equal to 1.05 (95% CI = 1.02–1.07) and [21] risk of death among the infant population is 1.04 (95% CI = 1.02–1.07) for an increase of 10 µg/m3 in PM_10_ concentrations [22].

However, to our knowledge no European meta-analysis was available to document more appropriate dose–response function to our geographic area.

There is clearly a need for further research to define the dose–response function more appropriate to the European shape of the risk function.

In addition, estimates of the burden of disease attributable to air pollution due to preterm birth complications and infant death are subject to a broad range of uncertainties [36]. Hereafter, we present a list of uncertainty sources that can be view as a call for improvement for future works.

- The combinations of multiple sources of input data that have each their own level of uncertainty.
- The assumptions choice in the applicability of the exposure or exposure–risk relationship to the country of concern.
- The multiple steps structuring the method, probably tend to accumulate the uncertainty.
- The few number of available studies assessing the risk of neurodevelopmental impairment among the preterm population, crucial information to estimate the DALYS.
- Potential biases in information include, for instance, the exposure–risk relationship derived from epidemiological studies or meta-analysis studies.
- Heterogeneity or validity of data sources for instance, the measure of exposure and health data.

Therefore, estimates of burden of disease have to be interpreted with caution due to complex assessment and wide of uncertainty that could affect the strength of the precision of estimate and then the formulation of accurate conclusions.

Secondly, we used all cause of infant death among all live birth without taking into account of sub-group (neonatal vs post-neonatal death) or specific causes of death. However, in the present study more appropriate data were not available at the census block level.

Thirdly, we hypothesized that all the newborns were exposed to the same PM_10_ value, ignoring both date and place of birth. The exposure level included in this risk assessment study is equal to the PM_10_ average in Paris over the study period. This could lead to misclassification of the exposure. It is known that both the number of births and the PM_10_ concentrations vary widely by season. The number of births is high during the spring and low during the last three months of the year. The three main PM_10_ emission sources in Paris are road traffic (28%), residential sector (26%) and “construction sites and quarries” (18%) [37]. During the summer, PM_10_ concentrations are lower, due to the slowdown of activities in Paris and the decrease in traffic that is associated with the holiday period. Taking into account the combination of the two sources of seasonality may affect the health consequences attributed to PM_10_ exposure.

Finally, despite the fact that the evidence supports increased neonatal morbidity and mortality for males across all gestation periods in comparison to females, we were unable to include sex-specific risk in the model because insufficient data was available. In addition, our study included too low a number of health events, which is why we did not stratify our analysis by time and space. This is also one reason why it was not possible to conduct in-depth analysis by neighborhood socioeconomic level.

Moreover, consideration of the dose–response function by socioeconomic class would be necessary to an examination of the well-documented interaction between air pollution and socioeconomic characteristics. However, no such data is available from the literature to perform a meta-analysis assessing a robust dose–response function per socioeconomic deprivation level: It is a call for additional environmental epidemiological studies investigating health consequences of air pollution exposure by socioeconomic class. The number of births is higher in the north-eastern part of Paris, where both air pollution and neighborhood socioeconomic deprivation are higher. Consequently, we expect to find a higher environmental burden of disease in this part of Paris, as has already been found for all-causes mortality [38].

## 5. Conclusions

From a public health perspective, with regard to maternal and child practice, individual-level interventions predominate. However, adverse birth outcomes result from a complex combination of individual determinants (including maternal behaviors during pregnancy) and the characteristics of the neighborhood in which the pregnant woman lives (including environmental nuisances). A healthy pregnant woman is more likely to have a healthy newborn; but a neonate born in a healthier place of residence (in terms of environmental exposure and social conditions) will tend to have better health trajectories throughout their life. Thus, it is essential to quantify health impacts related to environmental hazards for children at fine spatial scale, if we are to prioritize interventions. The French government is making efforts to provide medical care for pregnant women and to ensure good health for both newborn infants and mothers. However, our study suggests that additional effort is needed if we are to reduce the risk of complications and deaths associated with air pollution exposure among the preterm infants.

Indeed, our estimates suggest that PM_10_ may contribute substantially to the burden of preterm birth and infant death in Paris. Because of widespread exposure to PM_10_, significant health benefits could be achieved through regulatory interventions aimed at reducing exposure during pregnancy. Furthermore, the differential impact of air pollution by neighborhood socioeconomic level, though not statistically significant in our study, suggests that policymakers should consider the socioeconomic deprivation situation in their regulatory actions, in order to tackle the well-documented and longstanding disparities in terms of preterm births and risk of infant death.

## Figures and Tables

**Table 1:** Input data of the compartmental model for preterm birth born around the year 2010 (Blencowe et al., 2013 [24]).

Gestational Age	20–28 Weeks	28–32 Weeks	32–36 Weeks
Risks Estimates	% 95% CI	% 95% CI	% 95% CI
Case fatality risks (CFR)	28.3% (95% CI = 25.4–31.2%)	5.8% (95% CI = 5.1–6.5%)	0.7% (95% CI = 0.6–0.8%)
Risk of moderate/severe long-term neurodevelopmental Impairment	24.5% (95% CI = 20.2–28.8%)	12.2% (95% CI = 6.1–18.2%)	1.8% (95% CI = 1.5–2.3%)
Risk of mild long-term neurodevelopmental Impairment	33.9% (95% CI = 28.6–39.3%)	16.5% (95% CI = 6.1–18.2%)	3.4% (95%CI = 2.5–4.4%)

**Table 2:** Summary of input data included in the analysis.

Input Data/Parameters	Data Source/Reference	Descriptive
Concentration of PM_10_ between 2007 and 2012	The local association (AirParif)—using ESMERALDA Atmospheric Modeling system [18]	Mean 30.1 µg/m^3^ (Standard Deviation = 1.72 μg/m^3^)
Population size (the number of live births)	The birth certificate information registered by the Maternal and Child Care department of Paris	Total number of births = 86,877
Infant death over the period 2004–2009	Death certificates recorded at Paris City Hall	3.36 per 1000 live births
Preterm birth between 2009 and 2011	The birth certificate information registered by the Maternal and Child Care department of Paris	The rate of preterm birth reached 6.1%
**Age group**		
Preterm births among all births at less than 28 weeks and >20 weeks.	The birth certificate information registered by the Maternal and Child Care department of Paris	Rate of Preterm births equal to 0.22%
Preterm births among all births at 28–31 weeks	The birth certificate information registered by the Maternal and Child Care department of Paris.	Rate of Preterm births equal to 0.62%
Preterm births among all births at 32–36 weeks	The birth certificate information registered by the Maternal and Child Care department of Paris	Rate of Preterm births equal to 5.34%
**Dose–response function: Derived from meta-analysis**		
For Infant death	Rojas-Rueda et al. [22]	Risk of death among the infant population is 1.04 (95% CI = 1.02–1.07) for an increase of 10 µg/m^3^ in PM_10_ concentrations.
For preterm birth	Guo, et al. 2019 [21]	Increased risk of preterm birth related to an increase of 20 µg/m^3^ of PM_10_ concentrations is equal to 1.05 (95% CI = 1.02–1.07)
Disability weights	Blencowe, et al. 2013 [25]	For moderate-to-severe neurodevelopmental impairment: 0.38 (uncertainty range: 0.29–0.49). Assuming that about 50% of those with mild impairment had isolated mild problems, and 50% had mild motor and mild cognitive impairment, the disability weight used for these people was 10 fold lower, at 0.03 (uncertainty range: 0.02–0.05)

**Table 3:** Infant deaths by neighborhood socioeconomic level: Paris 2004–2009.

Neighborhood Socioeconomic Level	*N*	Death Rate per 1000 Live Births
1 (less deprived)	70	2.4
2	105	3.1
3	107	3.0
4	136	3.2
5 (most deprived)	205	4.5
TOTAL	623 ^&^	3.3
Ratio ^§^ (1/5)	1.9	

Legend: ^§^ the number of infant death living in the most deprived census blocks is reported on those living in the less deprived; ^&^ the census block is missing for 6 infant deaths.

**Table 4:** Preterm birth distribution by specific gestational age and by neighborhood socioeconomic level: Paris—2009–2011.

Neighborhood Socioeconomic Level	Extremely Preterm (<28 Weeks *)		Moderate Preterm (28–32 Weeks)		Late Preterm (32–36 Weeks)		TOTAL	
	*N*	%	*N*	%	*N*	%	*N*	%
1 (less deprived)	27	14.9	90	18.3	874	19.8	991	19.5
2	37	20.4	102	20.7	828	18.8	967	19.0
3	35	19.3	106	21.5	826	18.7	967	19.0
4	30	16.6	68	13.8	915	20.7	1013	19.9
5 (most deprived)	52	28.7	127	25.8	967	21.9	1146	22.5
TOTAL	181	100%	493	100%	4410	100%	5084 ^&^	100%
Ratio ^§^ (1/5)	1.92		1.41		1.11		1.16	

Legend: ^§^ the number of preterm birth living in the most deprived census blocks is reported on those living in the less deprived.* >20 weeks; ^&^ The census block information is missing for 3883 births including 279 preterm births.
